# Nano-Metal–Organic Framework Decorated With Pt Nanoparticles as an Efficient Theranostic Nanoprobe for CT/MRI/PAI Imaging-Guided Radio-Photothermal Synergistic Cancer Therapy

**DOI:** 10.3389/fbioe.2022.927461

**Published:** 2022-07-06

**Authors:** Yingjian Ma, Jing Mao, Haojie Qin, Pan Liang, Wenpeng Huang, Chenchen Liu, Jianbo Gao

**Affiliations:** ^1^ Department of Radiology, The First Affiliated Hospital of Zhengzhou University, Zhengzhou, China; ^2^ The First Affiliated Hospital, and College of Clinical Medicine of Henan University of Science and Technology, Luoyang, China; ^3^ School of Materials Science and Engineering, Zhengzhou University, Zhengzhou, China; ^4^ Forensic Medicine School of Henan University of Science and Technology, Luoyang, China

**Keywords:** nanoscale metal–organic framework, multi-modal imaging, photothermal, radiotherapy, theranostic nanoplatforms

## Abstract

The multifunctional theranostic nanoplatforms, which can realize changing the contrasts of medical images and enhance cancer therapies simultaneously, have attracted tremendous attention from chemists and medicine in past decades. Herein, a nanoscale metal–organic framework-based material was first synthesized and then decorated with platinum (NMOF545@Pt) successfully for multimodal imaging-guided synergistic cancer therapy. The obtained NMOF545@Pt is advantageous in shortening the longitudinal relaxation time (T1), enhancing photoacoustic effects, and elevating X-ray absorption efficiently. Thus, the enchantments of tripe imaging modalities, computed tomography (CT)/magnetic resonance imaging (MRI)/photoacoustic imaging (PAI), were realized with NMOF545@Pt administration simultaneously and can be cleared from the mice. Meanwhile, *in vitro* and *in vivo* experiments demonstrate that the synthesized NMOF545@Pt can dramatically increase photothermal therapy (PTT) and radiotherapy (RT) efficacy. Convincing evidence proves that tumor growth can be wholly inhibited without noticeable side effects or organ damage. The results demonstrated the promise of multifunctional nanocomposites NMOF545@Pt to improve biomedical imaging and synergistic tumor treatments.

## 1 Introduction

Cancer is still the leading cause of death in the world. The primary treatments for cancer include surgery, chemotherapy, and radiotherapy (Helmink et al., 2019). However, the existing methods are insufficient to restrain cancer and may have serious side effects (Demaria et al., 2019). To overcome this problem, significant efforts have been made. In the last decade, with the development of nanotechnology, nanomaterial-mediated antitumor treatments have shown great promise in various approaches (Chen et al., 2020a). For example, owing to the unique property of gold, nano-gold has been proven to enhance radiotherapy (RT), photothermal therapy (PPT), photodynamic therapy (PDT), and drug delivery (Jain et al., 2012; Beik et al., 2019). In addition, the therapeutic process and effects need to be monitored *in vivo* by medical imaging to determine whether the tumor has been completely inhibited. Computed tomography (CT) and magnetic resonance imaging (MRI) are the most common imaging techniques in modern medical tumor management. MRI is good at visualizing soft tissue abnormalities, while CT is adapted for bone-related diseases (Togashi, 2003; Schaefer and Langer, 2007). Furthermore, both CT and MRI can differentiate benign and malignant tumors by calculating and assessing the microcirculation perfusion of the contrast agent (CA) in the tumor and nearby regions (Wagner and Conti, 1991). Thus, it is critical and urgent to develop efficient theranostic nanoplatforms for imaging-guided cancer treatments (Park et al., 2009; Cheng et al., 2014).

Many efforts have been made to enhance the efficiency of tumor diagnosis and therapy in the last decades. Functional nanomaterials have become one of the most advanced branches and exciting forefront fields in biomedical engineering (Yu and Zheng, 2015). The conventional antitumor nanomaterials are inorganic nanomaterials, such as iron (Peng et al., 2008), gold (Shevchenko et al., 2008), silicon (Bertucci et al., 2019), and carbon (Zheng et al., 2016). Another strategy is depended on high polymer (Zhong et al., 2014), such as polydopamine and polypyrrole. Sometimes, the aforementioned two materials were incorporated into one nanocomposite to achieve highly efficient or multifunctional purposes (Lin et al., 2015; Liu et al., 2019). Alternatively, a metal–organic framework (MOF) is a class of compounds consisting of inorganic clusters and organic ligands. Thus, MOF can achieve multiple purposes with itself theoretically (Wu and Yang, 2017; Yang and Yang, 2020). Due to the unique features of MOF, the application has been spread over all fields: catalysis, gas storage, energy, wastewater treatment, and biomedicine (Ding et al., 2019; Chen et al., 2020b). As a new type of porous material, several nanoscale MOF (NMOF) has been tried to be used for cancer therapy by improving photothermal therapy (PTT), photodynamic therapy (PDT), radiotherapy (RT), and immunotherapy effects (Bieniek et al., 2021; Mallakpour et al., 2022). At the same time, the contrasts of multi-modality images can be enhanced with the administration of NMOF, such as MRI, CT, ultrasound (US), and photoacoustic imaging (PAI), which is meaningful for interventional therapy and monitoring disease progression (Wang, 2017; Bao et al., 2020; Liu et al., 2022). Additionally, benefitting from the abundant modification sites and stable structure, NMOF can be easily modified with a functional group for the synergetic enhancement. The folic acid (Yang et al., 2020a; Jiang et al., 2021) and hyaluronic acid (Xu et al., 2020) were added to the NMOF to provide tumor targeting ability, while the gold coating was used as enzymes for combined cancer therapy (Hu et al., 2020; Wang et al., 2022). It is highly expected that NMOF-based theranostic agents will play a more critical role in biomedical imaging and therapeutic applications (Lismont et al., 2017; Banerjee et al., 2020; Liu et al., 2021).

**SCHEME 1:**
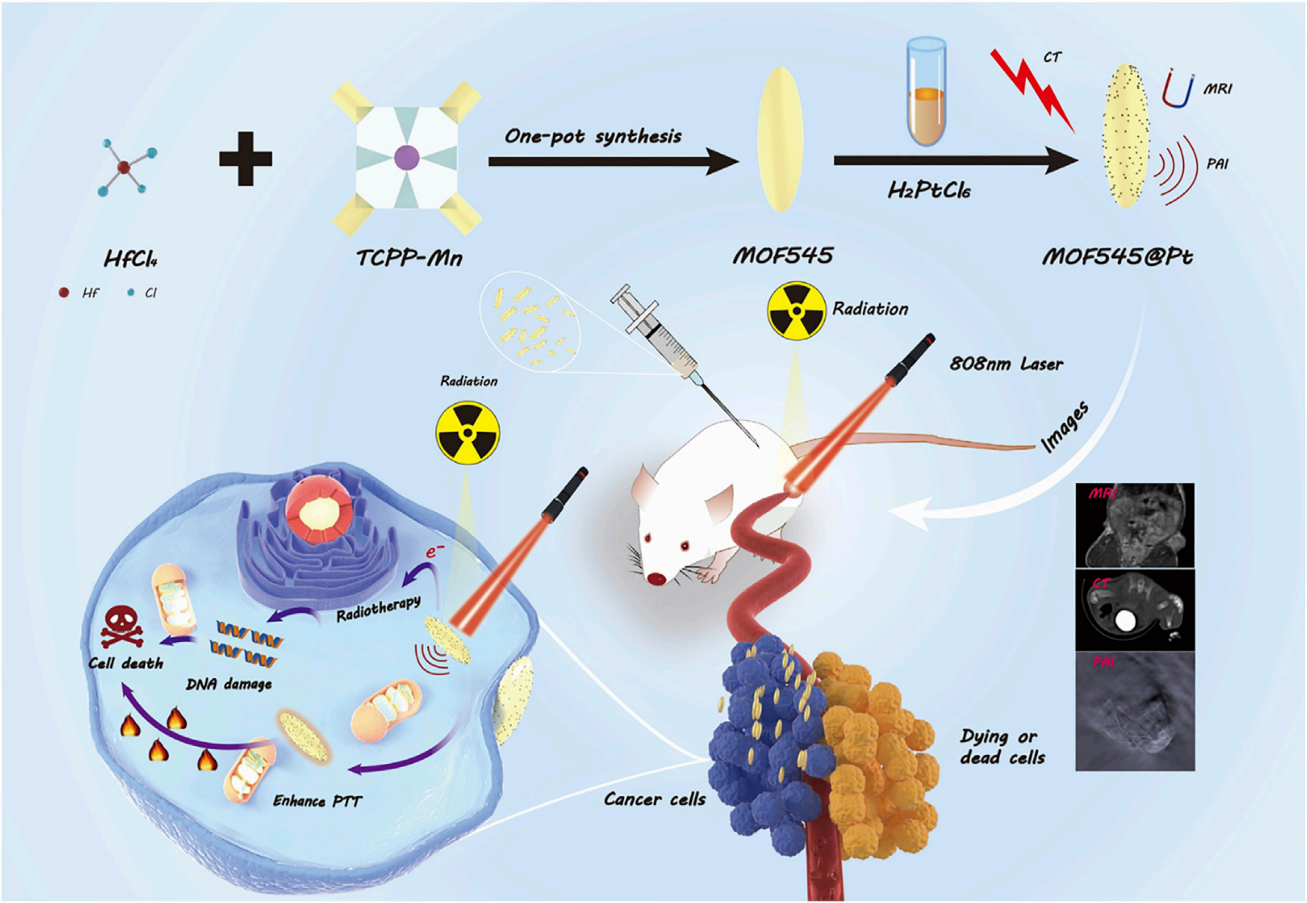
Schematic illustration of the preparation of NMOF545@Pt nanoparticles to integrate tumor CT/MRI/PAI imaging and PTT/RT.

As shown in Scheme 1, this study successfully synthesized MOF545@Pt by combing the two innately theranostic nanomaterials (NMOF545 and Pt-NPs). In this composite, the manganese in the center of the porphyrin can reduce the longitudinal relaxation time, and a T1W MRI image can be obtained, while the high-Z hafnium cluster and the platinum NPs work together to attenuate the X-ray penetration and CT images can be enhanced, and the RT can be achieved at the same time. The manganese porphyrin and the platinum NPs realize the photothermal conversion effect to realize the PTT purpose. Our *in vitro* and *in vivo* results demonstrated that the tumor growth could be completely inhibited by CT/MR/PAI imaging-guided synergistic RT-PTT therapy after being treated with NMOF545@Pt. Additionally, we did not observe apparent cytotoxicity or systemic toxicity by histological analysis in significant organs in a mouse, suggesting the excellent biocompatibility of NMOF545@Pt. Our results provide preliminary evidence that the MOF material-based multifunctional nanoparticles may open a novel avenue for achieving efficient tumor diagnostics and therapy.

## 2 Experimental Section

### 2.1 Materials

All materials and reagents in this study were obtained from several commercial suppliers. Benzoic acid (99%), pyrrole, HfCl_4_ (98%), MnCl_2_ (97%), and H_2_PtCl_6_▪6H_2_O (37.5%), thiazolyl blue (3-(4, 5-dimethylthiazol-2-yl)-2,5-diphenyltetrazolium bromide, MTT), were purchased from Aladdin Bio-Chem Technology Co. Limited (Shanghai, China). Ethanol, propionic acid, N, N-dimethylformamide (DMF, ≥ 99.8%), acetone, and ethylene glycol were purchased from Tianjin Chemical Reagent Company (Tianjin, China). 4T1 cancer cells were obtained from the college of pharmacy of Zhengzhou University. Cell culture medium Dulbecco’s modified eagle medium (DMEM), fetal bovine serum (FBS), and 0.25% pancreatin were purchased from Procell (Wuhan, China). Calcein acetoxymethyl ester (CAM), propidium iodide (PI), and 4′, 6-diamidino-2-phenylindole (DAPI) were purchased from Biyuntian, (Shanghai, China). All reagents or materials were used as received without further purification.

### 2.2 Sample Characterizations

Scanning electron micrographs (SEM) images and transmission electron microscope (TEM) images were obtained using JEOL JSM-6701F (JEOL Ltd., Tokyo, Japan) at 5.0 kV and JEOL JEM-2200FS (JEOL Ltd., Tokyo, Japan) at 120 kV, respectively. X-ray powder diffraction (XRD) spectra were collected on a D8 Advance diffraction spectrometer (Bruker Ltd., Germany). UV-vis absorption spectra were measured on a NanoDrop 2000 spectrophotometer (Thermo Scientific Ltd., United States) within a 190–840 nm wavelength range. (Thermo Scientific Ltd., United States) within the wavelength range of 190–840 nm. All samples’ hydrodynamic diameters and zeta potentials were measured on a dynamic light scattering (DLS) instrument (Malvern Zetasizer Nano-ZS90, Malvern Instruments, United States), and the results were averaged over three replaced measurements. The amounts of the elements were determined by an inductively coupled plasma atomic emission spectrometer (ICP-MS, Agilent 7500 CE, Agilent Technologies, United States).

### 2.3 Synthesis of MOF545 (Mn) NPs

Nanoscale MOFs were synthesized by a modified solvothermal method. The porphyrin organic linker (H_2_TCPP, meso-tetra(4-carboxyphenyl) porphine) was obtained by mixing the benzoic acid and pyrrole in the propionic acid and reflux overnight. Then hydrogen in the porphyrin center was replaced by manganese through the substitution reaction, and Mn-TCPP was obtained (Farokhi and Hosseini-Monfared, 2016). Then Mn-TCPP (150 mg) linker and HfCl_4_ (800 mg) were added to the DMF (150 ml) solution in a vial, and the mixture was sonicated for 15 min. Finally, the benzoic acid (800 mg) was added to the mixture, and the mixture was sonicated again for another 10 min. The vials were heated at 80°C in an oven for 48 h (Feng et al., 2012; Yu et al., 2020). After the reaction, the obtained product was washed with DMF ethanol and water several times until the supernatant became pellucid. Finally, the nano MOF545 powder was dried through freeze-drying and stored in dark conditions for the following use.

### 2.4 Synthesis of MOF545@Pt NPs

A polyol reduction technique was performed to synthesize the hybrid NMOF545@Pt composite (Guo et al., 2016). First, 200 mg of previously synthesized NMOF545 was added to a mixed solution with 2 ml aqueous and 8 ml ethanol in a beaker. Then the different amounts of H_2_PtCl_6_▪6H_2_O were added to the aforementioned mixture and stirred vigorously for 30 min. Then the beaker was heated to 60°C under stirring until obtaining the dry powder, which afterward was submerged in 20 ml ethylene glycol in another beaker. The beaker was then put in a water bath at a temperature of 95°C for 4 h under stirring. After the reaction cooling to room temperature, the solvent was discarded, and the remaining precipitates were freeze-dried.

### 2.5 Investigation of Imaging Enhancing and Photothermal Proprieties

#### 2.5.1 Measuring the Longitudinal (R1) and Transverse (R2) Relaxation Rates

All the MRI experiments were performed on a small-animal 7.0 T MR superconducting scanner (Preclinical Scan, MR Solutions Ltd., Guildford, United Kingdom) with an 8-channel coil for abdominal imaging only. The sample was dispersed in the water with different concentrations (0–0.4 mM [Mn]) in breakers and then transferred to 2 ml centrifuge tubes. Inversion recovery spin-echo pulse sequence was used to measure R1 with the following parameters: repetition time (TR) = 6,000 ms, echo time (TE) = 12 ms, inversion time changes from 20 to 2,000 ms (Wang et al., 1987), field of view (FOV) = 100 mm × 100 mm, slice thickness = 4 mm, and matrix = 128 × 128. According to the results of relaxation measurements, the coronal T1-weighted (T1W) images were obtained with optimized TR and TE. All medical image data was saved in DICOM format and analyzed using open-source ImageJ software (NIH, Bethesda, MD, United States)*.*

#### 2.5.2 Measuring the Computed Tomography Absorption Values

The CT absorption value of different samples was measured on a 16-slice CT scanner SOMATOM Sensation 16 (Siemens AG, Forchheim, Germany) with the following parameters: tube voltage = 80 kV, tube current = 200 mAs, slice thickness = 0.8 mm, and reconstruction index = 0.5 mm (Shi et al., 2016). Similar to the MRI phantoms, the CT phantoms were prepared in 2 ml tubes at different Hf concentrations from 0 to 50 mg/ml for NMOF545 and NMOF545@Pt.

#### 2.5.3 Assessing the Photoacoustic Effects

The enchantments of photoacoustic effects were assessed on a multispectral optoacoustic tomography (iThera Medical, Munich, Germany). The phantoms for PAI measurements were prepared as follows steps: first, samples with a concentration gradient of Hf (from 0 to 4 mg/ml), then the samples were put into a drinking straw with a diameter of 6 mm; finally, the straw was inserted in a gel sheath for scanning in the water bath. The imaging parameters including: wavelength = 800 nm and thickness = 2 mm (Meng et al., 2020a).

#### 2.5.4 Exploring the Photothermal Proprieties

The photothermal potentials of different materials were explored. First, the concentration and power effects were studied by dissolving NMOF and NMOF545@Pt (200 μg/ml) in PBS and then placed into PCR tubes for following infrared radiations (808 nm, 0.75 W/cm^2^, 10 min), respectively. Simultaneously, a thermal imaging camera recorded the maximum temperatures and the infrared thermals images (Testo 875–1i, Germany) every 30 s. Then, the thermal stability was accessed using a cyclic heating method on NMOF545 and NMOF545@Pt (200 μg/ml). For one circle, the sample was irradiated with a NIR laser (808 nm, 0.75 W/cm^2^), for 5 min, then cooled down for another 5 min naturally kept at environmental temperature (27°C ± 1°C). The thermal imaging camera recorded temperature alterations after repeating such a circle three times. The photothermal conversion efficiency was calculated (Chen et al., 2010):

$$\text{η}=\frac{{\text{Q}}_{\text{e}}}{{\text{Q}}_{\text{s}}}=\frac{\text{hS}\left({\text{T}}_{\text{max}}-{\text{T}}_{\text{min}}\right)-{\text{Q}}_{\text{dis}}}{\text{I}\left(1-10^{-{\text{A}}_{808}}\right)},$$ (1)

where *Q*_*e*_ and *Q*_*s*_ are the light power and water elevated power, *T*_*max*_ and *T*_*min*_ are final and original temperatures, *Q*_*dis*_ is the constant thermal parameters related to the solvent and container, *h* is the heat transfer coefficient, *S* is the surface area of the container, and *H*_*e*_ is constant (18.5 mW). *I* is the laser’s power density and *A*_*808*_ is the absorbance of samples at 808 nm.

### 2.6 *In Vitro* Experiments

The biocompatibility and the cytotoxicity triggered by the external stimulus of the synthesized NMOF545@Pt were studied with a standard MTT assay. 4T1 cells were cultured in DMEM media containing 10% FBS with 5% CO_2_ at 37 C (Medeiros et al., 2012). The cells were collected in the logarithmic phase with PBS and pancreatin. After low-speed centrifugation (1000 r, 3 min), the cells were resuspended in the PBS at a density of 5 × 10^5^. Then the 4T1 cells were transformed into 96-well plates at a density of 5 × 10^3^ and incubated for another 12 h before the following tests. Different concertation samples (0, 25, 50, 100, 200, and 400 μg/ml) were incubated with cells for 12/24 h, and then 100 μl of MTT was added to each well before reading by an ELISA reader at 570 nm. Radiotherapy was performed on an image-guided small animal irradiator (X-RAD 225, precision X-ray, United States). In order to observe the live or dead cell viability, classic CAM/PI double staining (green-red) was performed as well as previously reported (Yang et al., 2020b).

### 2.7 Animal Models

Three-week-old female BALB/c mice were purchased from Beijing Huafukang Bioscience (Beijing, China) and kept in specific pathogen-free conditions at the Laboratory Animal Center of the First Affiliated Hospital of Zhengzhou University. All mice experimental procedures were approved by the Animal Ethics Committee of Zhengzhou University. Tumor-bearing mice were prepared by subcutaneously injecting 50 μl 4T1 cells (2 × 10^6^) into the right hind. After about 1 week, the tumor volume reached about 120 mm^3^ (day 0), and then the mice were then divided into different groups for following imaging and treatment studies. 200 μl samples (10 mg/kg) were injected into the tumor-bearing mice by intravenous injection from the tail vein before experiments. According to previous similar studies, the time points for imaging are predetermined (Meng et al., 2020b; Wang et al., 2020).

### 2.8 *In Vivo* Synergistic Cancer Therapy

Twenty-four tumor-bearing mice were divided into four groups randomly: 1) NMOF545@Pt; 2) NMOF545@Pt+RT; 3) NMOF545@Pt+PTT; 4) NMOF545@Pt+PTT+RT. The treatments were repeated the day after the first treatments to enhance the therapy efficiency. The length and width of tumors and body weight were recorded every 2 days for 2 weeks after various treatments. The tumor volume was calculated as follows: width^2^ × length/2. After the second treatment, one mouse in each group was sacrificed, and major organs were excised for histological examination (hematoxylin and eosin, H&E). Additionally, the blood samples were collected for blood biochemistry tests to reconfirm the biocompatibility of NMOF545@Pt NPs further.

### 2.9 Statistical Analysis

All the analyses were performed in SPSS 16.0 software (Chicago, United States) for Windows. The results obtained with several time measurements are reported in mean and standard deviation (SD) format. The differences between different groups are analyzed using the Student’s t-test and *p* < 0.05 is considered statistically significant.

## 3 Results and Discussion

### 3.1 Synthesis and Characterization of NMOF545@Pt NPs

**FIGURE 1:**
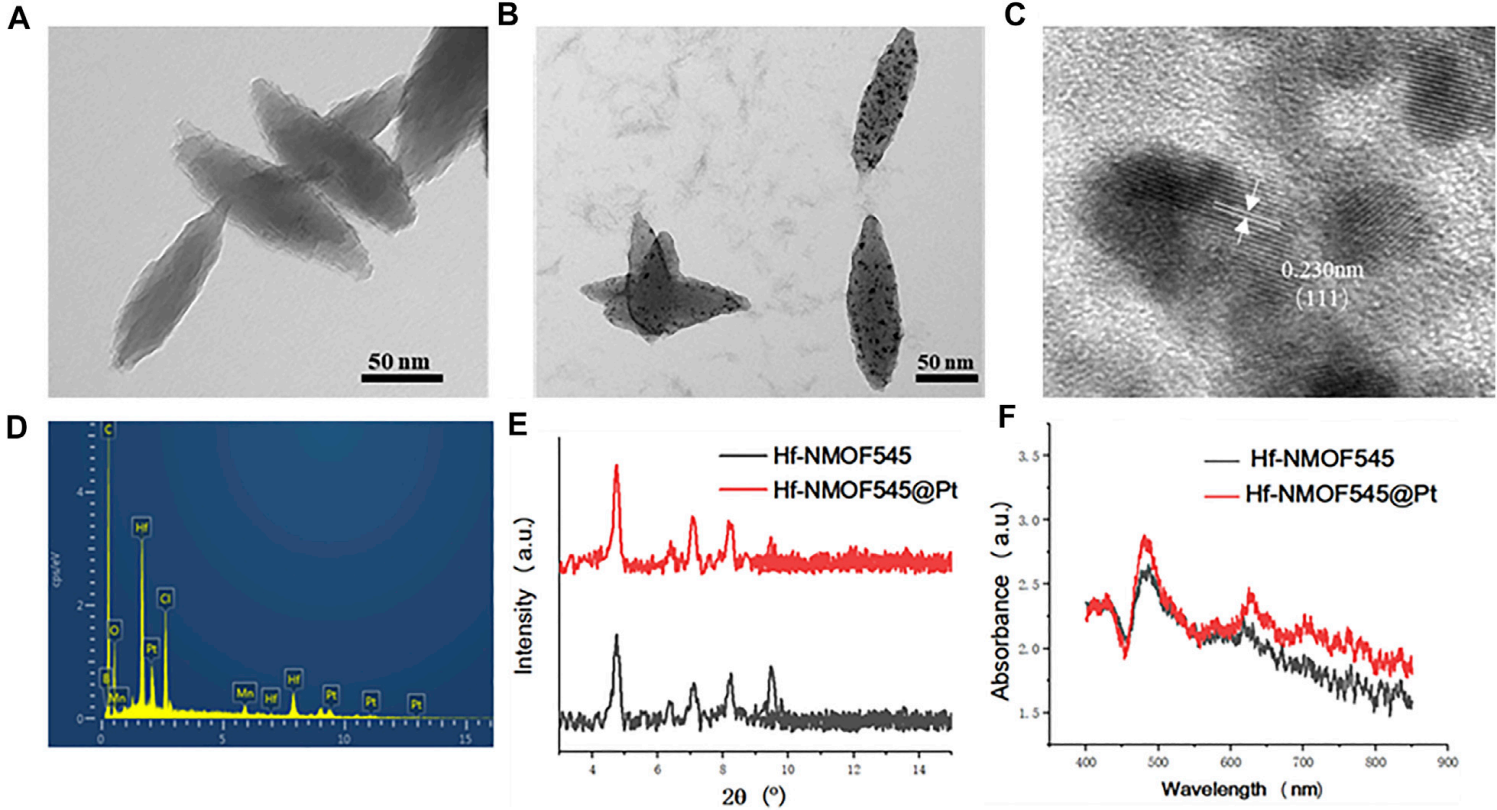
Characterization of NMOF545 and NMOF545@Pt nanocomposites. **(A)** TEM image of NMOF545; **(B)** TEM image of NMOF545@Pt nanoparticles; **(C)** high-resolution TEM image of Pt NPs; **(D)** EDS spectrum of NMOF545@Pt nanoparticles; **(E)** XRD patterns of the NMOF545 and NMOF545@Pt; **(F)** UV-vis absorption of NMOF545 and NMOF545@Pt.

**FIGURE 2:**
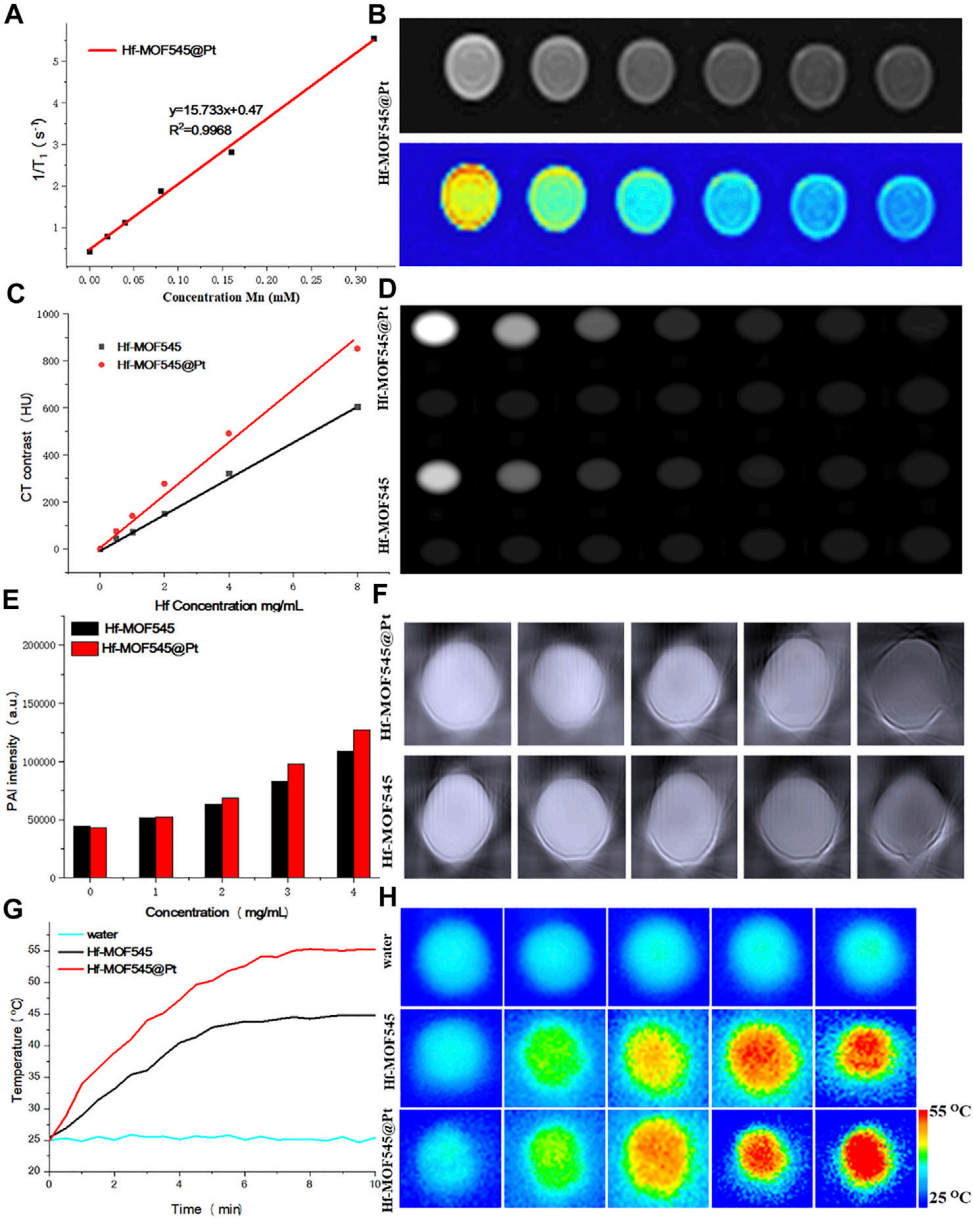
*In vitro* MRI/CT/PAI characterization of NMOF545 and NMOF545@Pt. **(A)** T1 relaxation rates of NMOF545@Pt and T1-weighted MR images **(B)**; **(C)** the HU value of NMOF545 and NMOF545@Pt nanocomposites against the Hf concentration and CT images of different concentrations of nanocomposites **(D)**; **(E)** PAI signal intensity changes with the increasing concentrations and the PAI images of two samples **(F)**; **(G)** temperature increasing profiles of water, NMOF545 and NMOF545@Pt (200 μg/ml) using 808 nm NIR laser powers (0.75 W/cm^2^) for 10 min; and **(H)** thermal images of different samples for different durations.

As shown in Figure 1, the NMOF545 was first synthesized according to previous studies (Yu et al., 2020), and then the ultra-small platinum partials were loaded on its surface (Meng et al., 2020b). SEM and TEM measured the morphologies of the NMOF545 and NMOF545@Pt. As shown in Figure 1A, the NMOF545 shows a spindle ship, the same as previous studies (Feng et al., 2012), with 111 ± 24/35 ± 11 nm for the long/short diameter, respectively. Moreover, the SEM images of the two samples are shown in Supplementary Figure S1, and uniform rice-like nanorods were further confirmed. After being decorated with platinum partials, tiny black dots show up, as shown in Figure 1B, and the diameter of Pt NPs is around 1–4 nm. As shown in Supplementary Figure S3, the amounts of black dots can be easily controlled by increasing the H_2_PtCl_6_ content from 5, 10, 20, and 40 mM with 100 mg NMOF545 in the initial solutions. The HRTEM image in Figure 2C clearly shows that the lattice spacing of Pt NPs is 0.230 nm, which agrees with the (111) plane. It should be noticed that the Pt peak was not observed in the XRD result, which can be attributed to the small size of the Pt NPs. Furthermore, the EDS spectrum confirmed the uniform distribution of three critical elements, including Hf, Mn, and Pt, as shown in Figure 1D. Moreover, the mount of the Pt was further analyzed by ICP-MS: 1.63, 3.11, 5.99, and 11.83 wt%, respectively, for 5–40 mM H_2_PtCl_6_. In addition, as shown in Figure 1E, the synthesized NMOF and NMOF545@Pt were further re-confirmed by XRD. It should be noted that both samples show almost the same spectra patterns as that of NMOF545, and no apparent difference was observed, as mentioned earlier. Dynamic light scattering revealed that the NMOF and NMOF545@Pt NPs had positively charged surface potentials of 18.5 ± 3.2 eV and 6.2 ± 2.1 mV, with average hydrodynamic diameters of ∼181 and ∼215 nm, respectively (Supplementary Figure S2). In addition, the UV-vis spectra were used to confirm the successful Pt loading. Compared with the NMOF545, the NMOF545@Pt shows an elevated absorption envelope around 800 nm, as shown in Figure 1F, which indicates the potential to enhance photothermal effects.

### 3.2 Enhancing Multi-Modality Imaging Properties

Due to the high R1 and low biotoxicity, manganese (III) porphyrins have drawn considerable attention recently as a positive contrast agent for reducing the longitudinal time (T1). Thus, the pre-designed NMOF545@Pt has a unique manganese porphyrin as the organic linker and is expected to change the MRI image contrasts. As shown in Figure 2A, the R1 is measured against the concentration of Mn, and the value is 12.64 mM S^−1^, which is large enough for changing the MRI contrasts at a relatively low and safe concentration. The T1W images directly show that the images of the NMOF545@Pt phantom are brighter than water only phantom, and the brightness increased with the dose of the sample, as shown in Figure 2B. The imaging results further confirmed the ability of the obtained NMOF545@Pt as a potential MRI contrast agent. The phantoms explored the X-ray attenuation (CT) properties of the synthesized NMOF545 and NMOF545@Pt with various concentrations. As shown in Figures 2C,D, along with the increase of the concertation, the phantom becomes brighter, and obviously, contrast can be observed at 50 mg/ml Hf compared with water. The Hounsfield unit (HU) value increased with concentration for all samples. The HU values were plotted against the concentration of Hf, and the enhancement effects show a good linear dose-dependent manner. Importantly, compared with the naked NMOF545, the Pt-loaded sample boosted the HU values with the same Hf concentrations, which is attributed to the combined impacts of the two high-Z elements. Moreover, the board absorption in NIR widow makes the NMOF545@Pt an attractive candidate for use as PAI contrast agents. As shown in Figures 2E,F, NMOF@545@Pt has excellent enchaining PAI capability as well, and the brightness of PAI images increased with increasing the concentrations of the samples (Figure 2B). Moreover, NMOF545@Pt shows a stronger PAI contrast-enhancing ability than NMOF545 at the same concentration. These results preliminary demonstrated that the synthesized NMOF545@Pt could shorten the T1 relaxation time, converse the laser pulse into heat and efficiently absorb X-rays. It also should be noticed that the performance of NMOF545@Pt as the contrast agent is equal to or better than that of NMOF545. Thus, the NMOF545@Pt may hold great potential to simultaneously satisfy the needs of enhancing CT, MRI, and PAI triple modality images.

### 3.3 Photothermal Performance in Solution

The high NIR absorption of NMOF545@Pt inspired us to study the photothermal effect with the 808 nm laser irradiation (0.75 W/cm^2^, 10 min). As shown in Figures 2G,H, NMOF@545@Pt (200 μg/ml) temperature has increased from ∼25.4°C to ∼57.6°C. In marked contrast, the NMOF545 (200 μg/ml) only increased from ∼25.5°C to ∼45.5°C, and no apparent temperature changes were observed for the pure water phantom. An IR camera records the temperature changes, and the photothermal conversion efficiencies were also calculated for both samples with the same laser on/off conditions, as shown in Supplementary Figure S4. After three laser on/off cycles, the temperature can still reach higher than 55°C after three on/off laser cycles, indicating good photothermal stability. The linear relationship was plotted between the negative natural logarithm of falling temperatures and time, 44.64% for NMOF and 55.7% for NMOF545@Pt, respectively (Supplementary Figure S5). All these results proved that the synthesized NMOF545@Pt has the potential to serve as an efficient PTT agent.

### 3.4 *In Vitro* Cell Experiments

**FIGURE 3:**
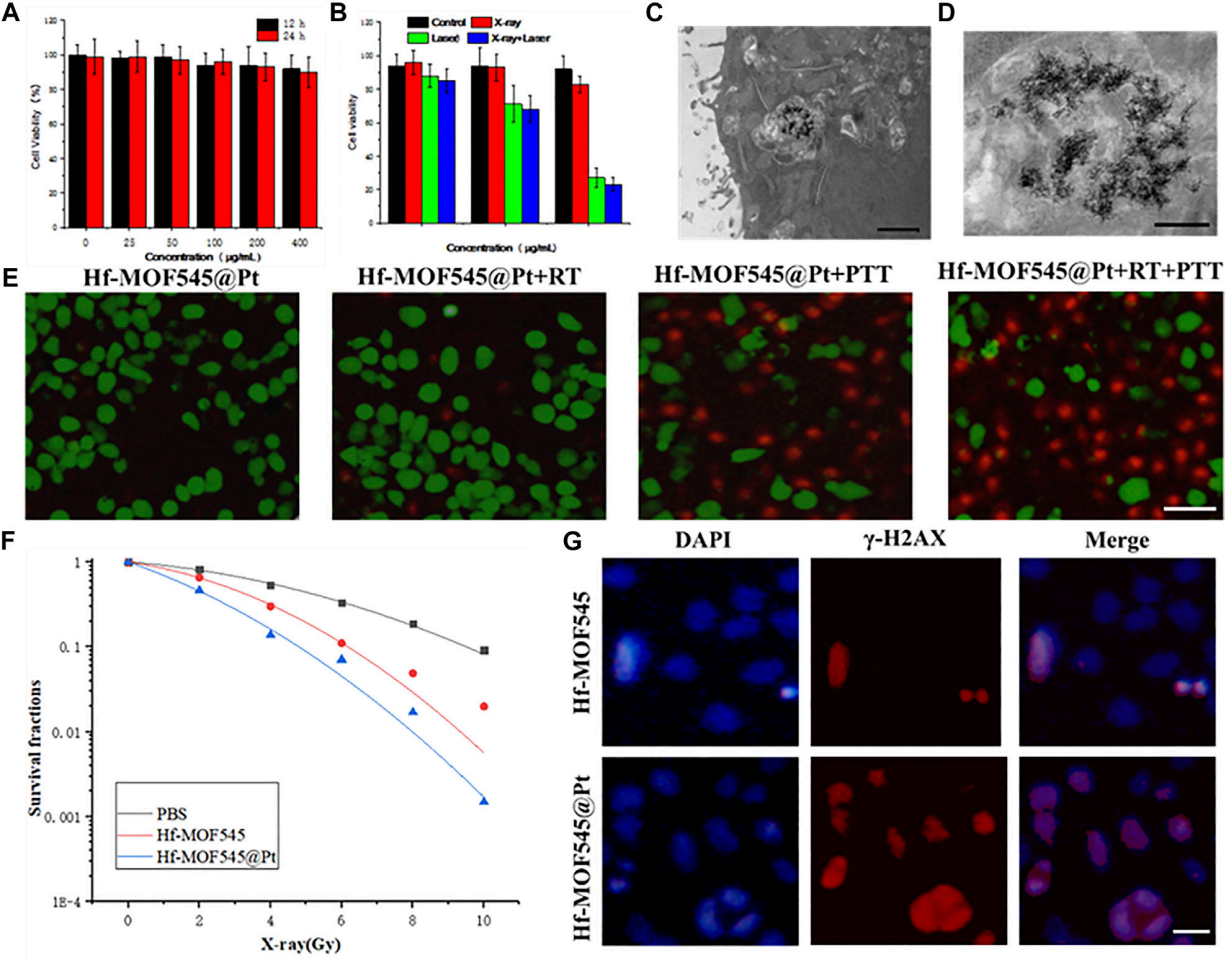
*In vitro* cytotoxicity experiments. **(A)** 4T1 cells viabilities after incubation with different concentrations of NMOF545@Pt for 12 and 24 h; **(B)** 4T1 cells viabilities after different treatments with different concentrations of NMOF545@Pt: NMOF545@Pt, NMOF545@Pt + laser (0.75.0 W cm^−2^, 10 min), NMOF545@Pt + X-rays (4 Gy) and NMOF545@Pt + laser + X-rays; **(C)** and **(D)** are representative TEM images of nanoparticles entrapped in vesicles of 4T1 cells after 12 h of co-cultured with NMOF545@Pt, and the scale bar is 1 and 0.5 μm for **(C)** and **(D)**, separately; **(E)** representative survival images of 4T1 cells after various treatment with NMOF545@Pt under PTT/RT. The green fluorescence represents live cells, and the red fluorescence represents dead cells, and the scale bar is 20 μm; **(F)** radiation dose-survival curves of 4T1 cells cultured with different samples; **(G)** immunocytochemical analysis of γ-H2AX expressed in 4T1 cells and nucleus were stained with an anti-γ-H2AX antibody (red) and DAPI (blue) 24 h after RT, and the scale bar is 10 μm.

To investigate the therapeutic effect of the NMOF545@Pt, cytotoxicity tests were performed with 4T1 cells using the standard MTT method. After incubation with NMOF545@Pt at different concentrations for 12/24 h, the cell viabilities were maintained at a high level, as shown in Figure 3A. It indicated t no apparent cytotoxicity in both samples, even at a very high concentration (400 μg/ml). The excellent biocompatibility is a prerequisite for further animal studies and an essential condition for modern biomedicine. The tumor cell-killing ability triggered by the 808 nm laser and X-ray was also explored using a standard MTT assay. As shown in Figure 3B, after the irradiations of 808 nm laser and 4 Gy X-ray, the cell viability drastically decreased, and cytotoxicity increased along with the NMOF545@Pt concertation increasing. Figures 3C,D (magnified) show that the cellular uptake by 4T1 cancer cells of NMOF545@Pt could be confirmed by TEM images. Moreover, live/dead co-staining by the calcein-AM/PI was used to check cell viability visually. As shown in Figure 3E, the cells treated by NMOF545@Pt without any treatments exhibit almost whole green fluorescence (live) and rarely red fluorescence (dead). While, after different irradiations, the cells incubated NMOF545@Pt exhibited numerous red fluorescence, which means the cells were killed successfully by external stimulus. The strongest red fluorescence was observed in the combination therapy of PTT and RT, indicating the maximum antitumor efficacy. In addition, to evaluate the radiotherapy efficacy at the cellular level, the clonogenic assays and the surviving fraction curve were shown in Figure 3F. Additionally, the γ-H2AX assays and colony formation experiments were performed to verify the RT-induced DNA damage in cells and the NMOF545 and NMOF545@Pt as effective radiosensitizers. As shown in Figure 3G, weak red fluorescence (γ-H2AX) but strong nucleus blue fluorescent (DAPI) was observed in the NMOF545 group. More red fluorescence was generated in the nucleus regions for the cells treated with NMOF545@Pt, indicating the double-strand DNA breaks. Together, these results manifest that the NMOF545@Pt can enhance PTT and RT’s efficacy in inhibiting the tumor cells.

### 3.5 *In Vivo* Multi-Modality Imaging Enhancements

**FIGURE 4:**
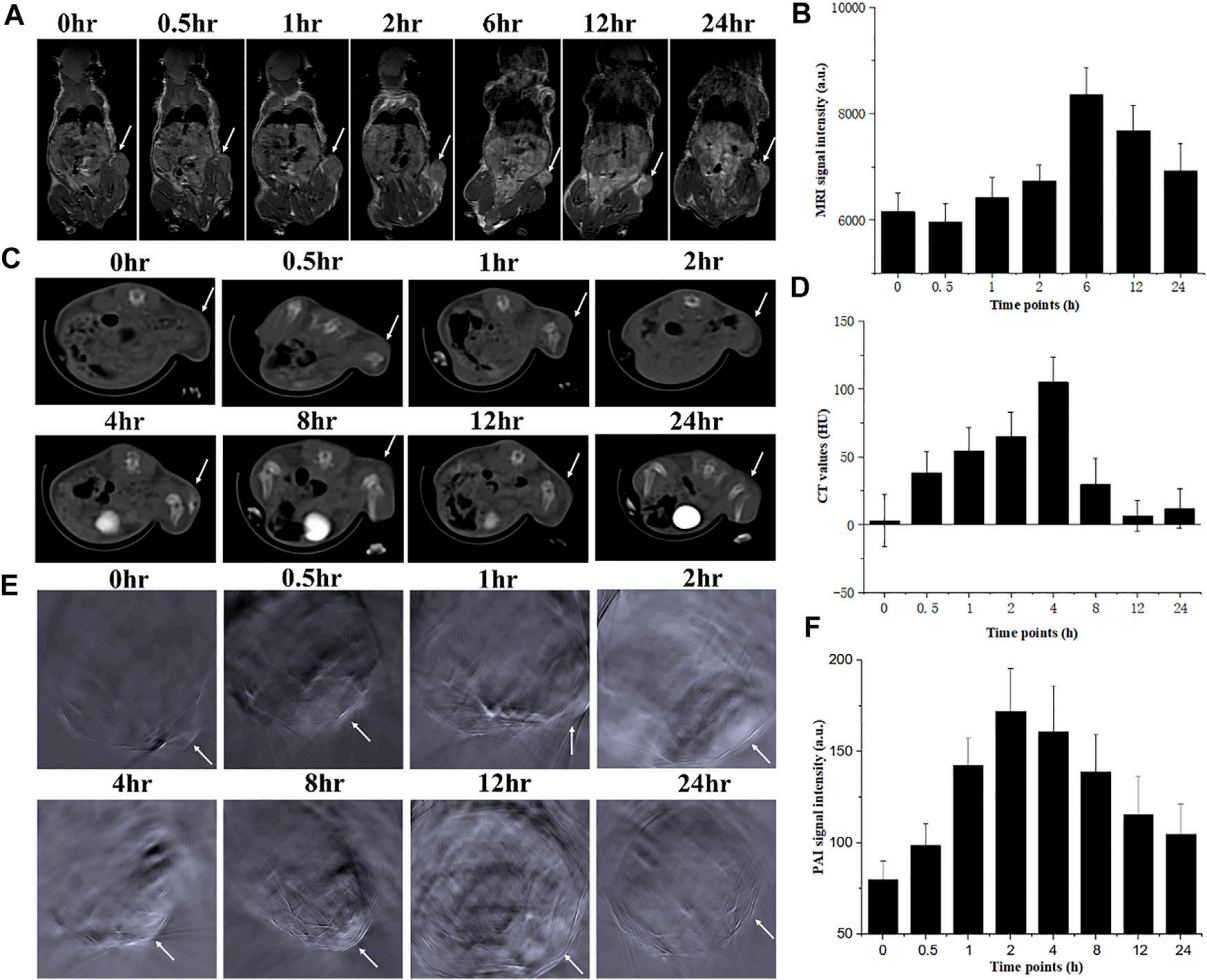
*In vivo* dynamic triple images of NMOF5454@Pt in the 4T1 breast tumor mouse model and relative signal increments at different time points for 24 h. **(A)**, **(B)** Coronal MRI images; **(C)**, **(D)** axial CT images; **(E)**, **(F)** axial PAI images. White arrows denoted the tumor sites.

Encouraged by the excellent properties of changing the contrast of CT/MRI/PAI images in the phantom, the *in vivo* imaging enhancing behaviors of NMOF545@Pt were further investigated by triple modality imaging methods on 4T1 tumor-bearing mice. In the *in vivo* MRI study, as shown in Figure 4A, 100 µl 1 mg/ml NMOF545@Pt dissolved in the saline was intravenously injected into the mouse from the tail vein. Compared with the image before injection, we can see that the MR signal intensity of the tumor region remarkably increased on T1W images, which means the NMOF545@Pt has been enriched in the tumor site through the blood circulation. The changing cure of the mean signal intensity was plotted, and the maximum enhancement appeared after injection of 6 h and then fell. In the *in vivo* CT scan, as shown in Figure 4B, it is hard to distinguish the tumor edges from the nearby muscles. This is because both tissues have very closed HU values. As expected, after administration of the NMOF545@Pt, the tumor site brightened, indicating the samples’ accumulation. From the mean HU changing curve (Figures 4C,D), we can see that the maximum CT signal intensity appeared after injection of 4 h and then fell. A similar signal intensity change pattern was observed in the *in vivo* PAI study, as shown in Figures 4E,F. As previously mentioned, the tumor-bearing mouse was scanned at a wavelength of 860 nm before and after the injection of NMOF545@Pt. We found the PAI signal intensity increased with time and reached its maximum in the tumor site after injection of 2–4 h, the same as the CT imaging. The quantitative analysis of the signal change cure is consistent with that of CT. The time point of maximum values for different imaging modalities is different, especially for MRI. This is because the high concentration of the Mn can compromise the enhancement of the T1 signal, thus the time point for the highest accumulation of sample should be 6 h after injection, which can be confirmed from the CT results. The *in vivo* imaging results reconfirmed that NMOF545@Pt as a contrast agent for triple imaging modality has great promise to be used in future diagnostic imaging.

### 3.6 Synergistic Cancer Therapy

**FIGURE 5:**
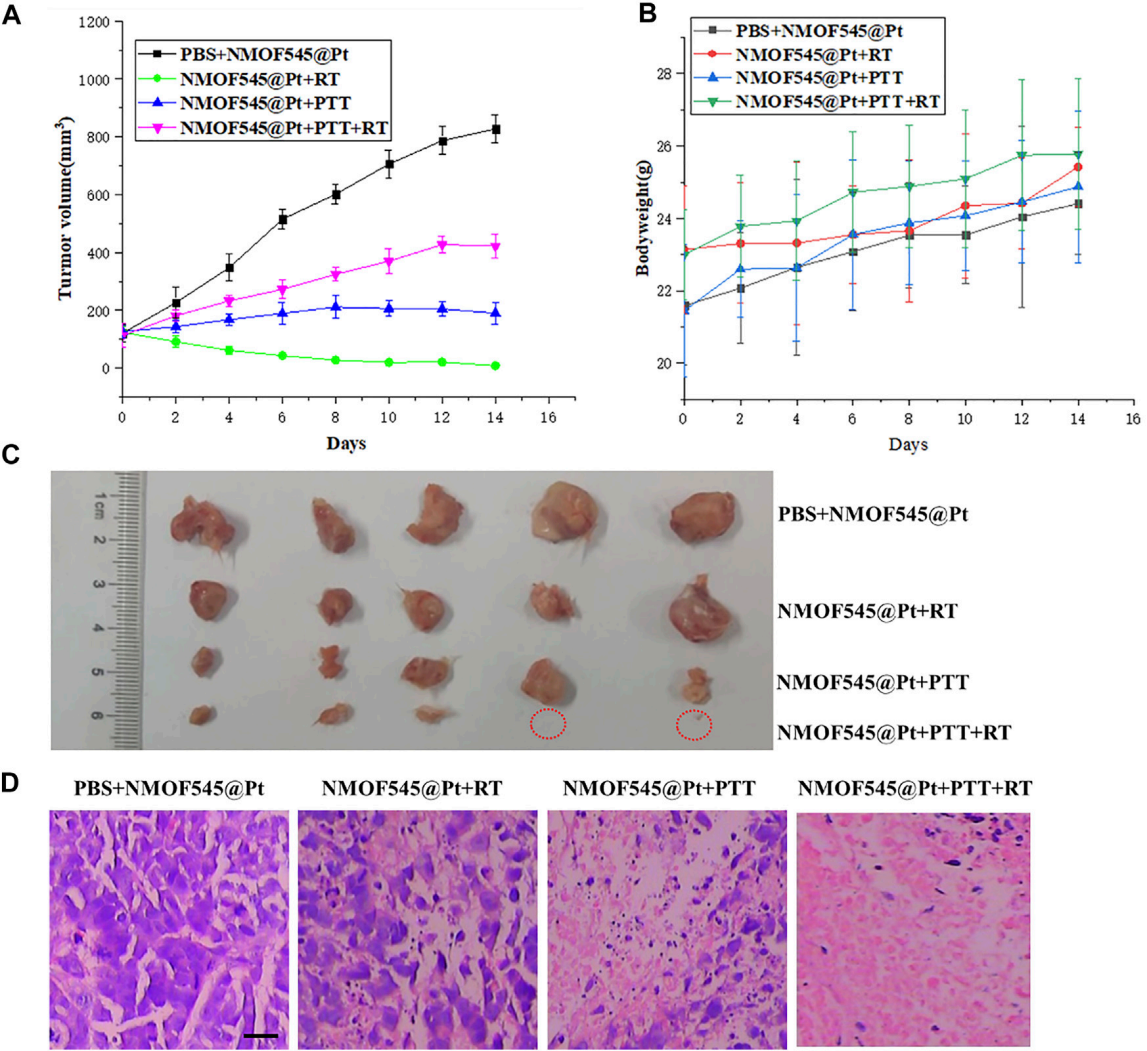
*In vivo* tumor therapy efficiency of NMOF545@Pt nanoparticles. **(A)** Bodyweight and tumors volume. **(B)** Changing curves of 4T1 tumor-bearing mice at different time points after various treatments. **(C)** Photographs of all excised tumors in mice were sacrificed at the end of observation and the red circles denoted the disappeared tumors. **(D)** H&E staining of tumors shows the organizational structure in different groups (scale bar, 100 μm).

Next, the feasibility of NMOF545@Pt for *in vivo* synergistic cancer therapy was further studied. As described previously, 4T1 tumor-bearing mice were randomly divided into four groups (*n* = 6) for different treatments as follows: 1) PBS+NMOF545@Pt, 2) NMOF545@Pt + RT, 3) NMOF545@Pt + PTT, and 4) NMOF545@Pt RT + PTT. PTT was conducted with the 0.75 W cm^−2^, 808 nm laser, and RT was conducted with a 4 Gy X-ray radiation dose. Figure 5A shows the tumor volume changes of different groups. Clearly, all the treatment groups show significantly smaller values than the PBS control group without any interventions, showing the tumor growth vigorously. Moreover, the tumor volume in the combined therapy group, NMOF545@Pt + PTT + RT, was suppressed, while the other three treatment groups show only partly inhibited compared to the PBS group. In addition, as shown in Figure 5B, we did not find significant weight loss for all groups, which further confirmed the good biocompatibility of NMOF545@Pt with fewer side effects. After two weeks, the mice were sacrificed and the tumors were excised as shown in Figure 5C, in which the tumors for combination therapy were significantly inhibited. Moreover, all indexes of blood biochemistry (Supplementary Table S1) were within the normal range and no significant differences were observed between the sample group and the other two control groups. Meanwhile, as shown in Figure 5D and Supplementary Figure S5, the H&E staining was used to assess the damage to the tumors and major orangs caused by the whole treatment. The tumors in the treatment groups show varying degrees of necrosis, and the NMOF545@Pt RT + PTT group almost disappears of living tumor cells, consisting of the volume measurement results. While no obvious cell damage could be observed in five major organs for all groups. Obviously, the *in vivo* therapeutic experiments confirm that the NMOF545@Pt is biocompatible and promising to be used as efficient nanotheranostics agents*.*

## 4 Conclusion

In conclusion, we have fabricated a multifunctional MOF-based nanocomposite integrated with NMOF545 and Pt for enhancing triple imaging modality CT/MRI/PAI guiding tumor PTT and RT synergistic therapy. In this theranostic nanoplatform, the enhancement of T1W MRI imaging was realized by the Mn in the porphyrin, and the NIR absorption character could be attributed to Pt, and the porphyrin and the capacity of attenuate X-ray is profit from the high-Z elements Hf and Pt. Furthermore, we found that photothermal and RT effects can be simultaneously improved after loading Pt nanoparticles. The *in vitro* and *in vivo* results highlight the significant potential of NMOF545@Pt as a multimodal contrast agent and highly effective therapeutic agent, which may facilitate clinical outcomes in the future.

## Data Availability

The original contributions presented in the study are included in the article/Supplementary Material; further inquiries can be directed to the corresponding author.
