# Supplementary material for: Nano-Metal–Organic Framework Decorated With Pt Nanoparticles as an Efficient Theranostic Nanoprobe for CT/MRI/PAI Imaging-Guided Radio-Photothermal Synergistic Cancer Therapy
Source: Front Bioeng Biotechnol. 2022 Jul 6;10:927461. doi: 10.3389/fbioe.2022.927461 (PMC9298652; doi:10.3389/fbioe.2022.927461)
Supplement: Supplementary file 1 [file DataSheet1.docx]

Supplementary Information

**Nano metal-organic framework decorated with Pt nanoparticles as an efficient theranostic nanoprobe for CT/MRI/PAI imaging-guided radio-photothermal synergistic cancer therapy**

1. Representative SEM images of NMOF545 (A) and NMOF545@Pt (B) (scale bar, 50 nm).


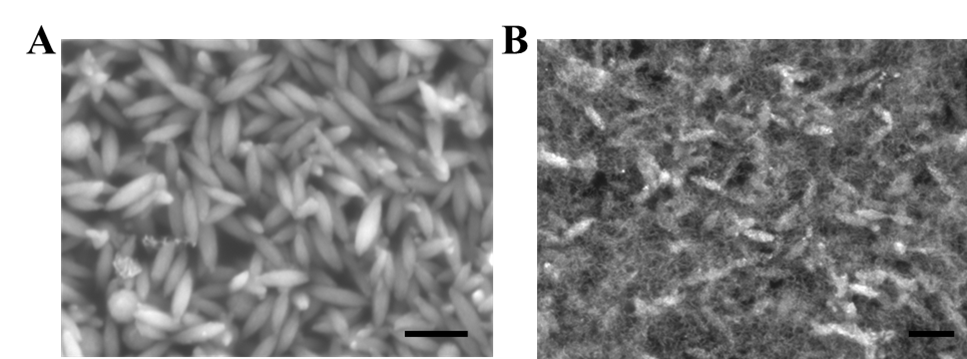


1. TEM images of NMOF545@Pt with different ratios between H_2_PtCl_6_▪6H_2_O and NMOF545 (scale bar, 50 nm). .


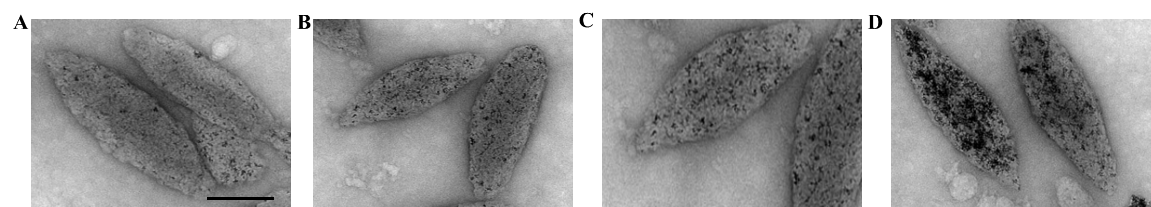


1. Zeta potential of NMOF545 and NMOF545@Pt.


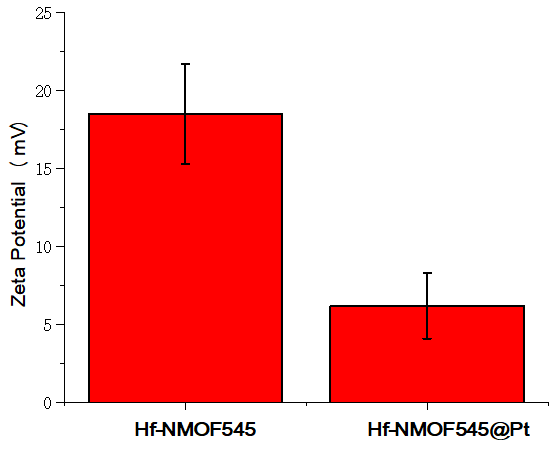


1. Temperature cycles of the solution containing NMOF545@Pt 200 µg mL⁻¹ with 808 nm laser ON/OFF irradiation.


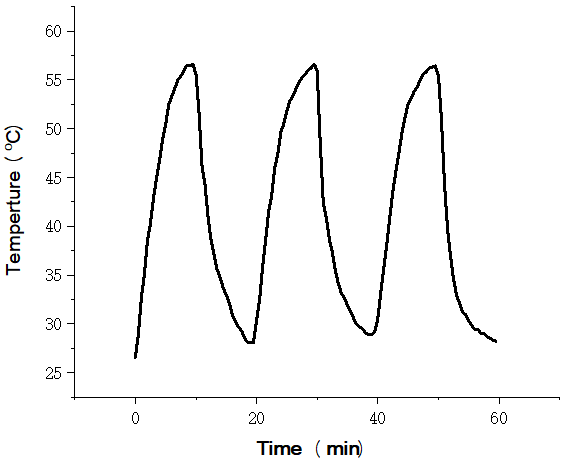


1. Photothermal effect calculation by linear fitting time versus negative natural logarithm of driving force temperature of NMOF545 (A) and NMOF545@Pt (B).


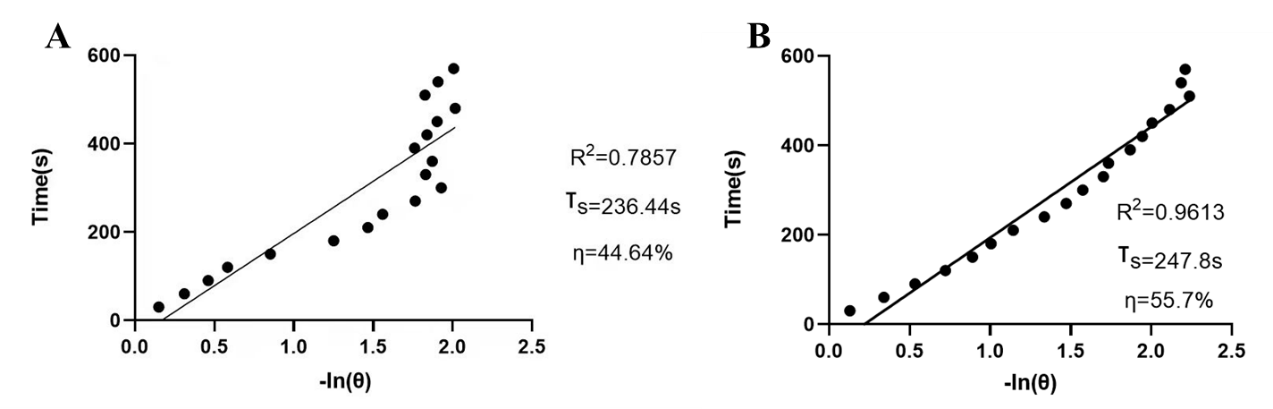


1. Hemocompatibility of NMOF545@Pt nanoparticles. Mouse blood was incubated with samples for 2 h at 37°C


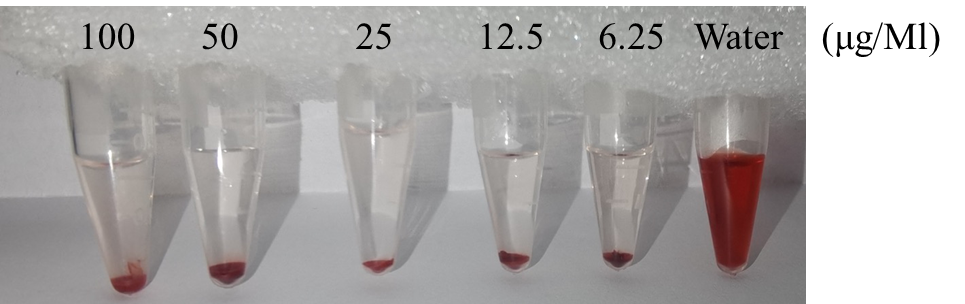


1.
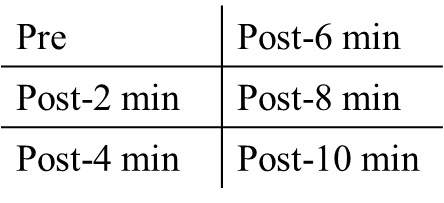

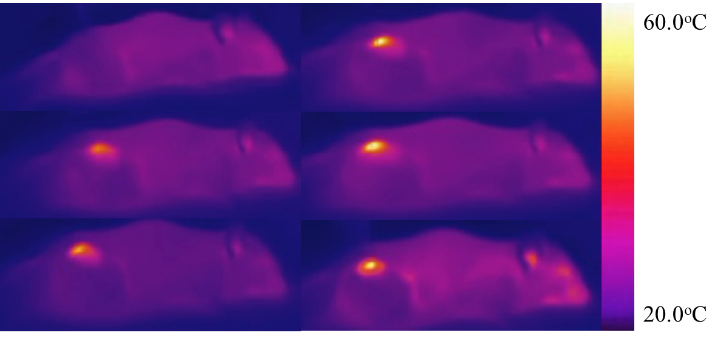
In vivo thermal images of 4T1 tumor-bearing mice treated with NMOF545@Pt, and then the tumor was irradiated by to 808 nm laser.
2. The histological images of one mouse main organs (heart, liver, spleen, lung, and kidney) for different treatments (scale bar, 100 μm).


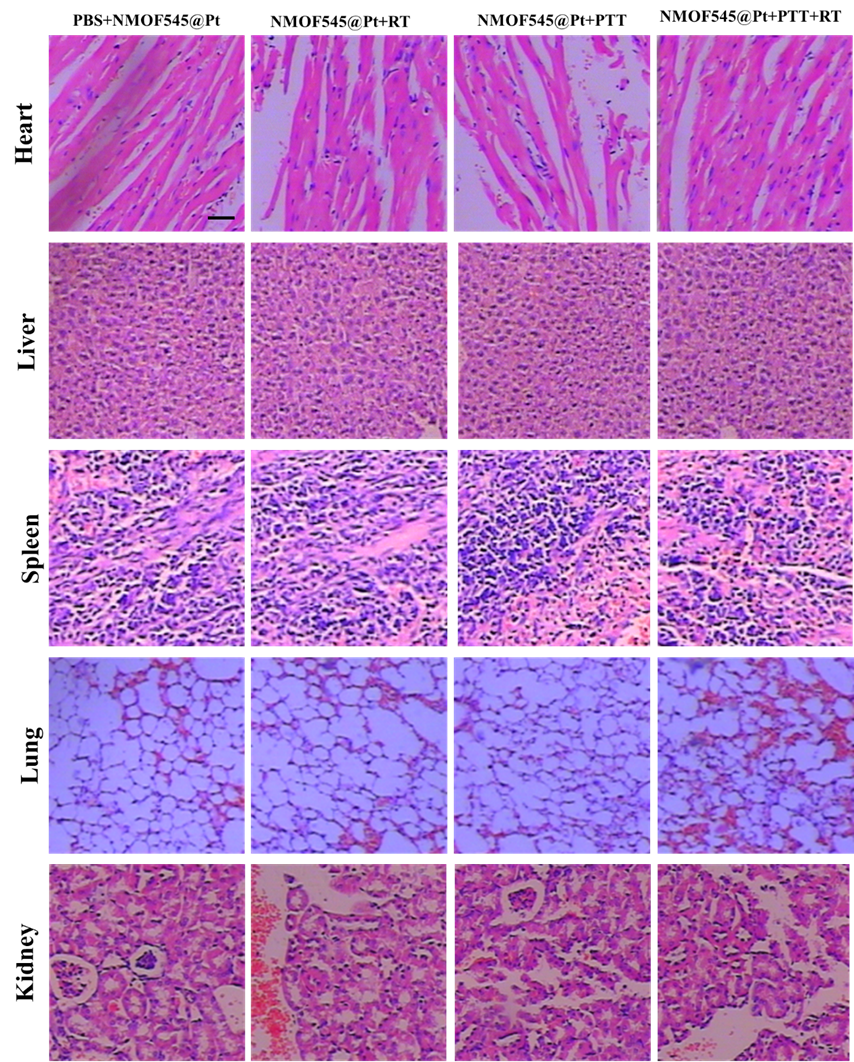


1. The blood biochemistry of different samples.

Table S1.

| Name | Units | Control | PBS | NMOF-545@Pt |
| --- | --- | --- | --- | --- |
| WBC | 10^9^/L | 3.84 ± 1.12 | 3.51 ± 0.66 | 4.04 ± 0.85 |
| RBC | 10^12^/L | 10.08 ± 0.44 | 10.55 ± 0.24 | 9.97 ± 0.72 |
| HCT | % | 50.03 ± 3.2 | 48.84 ± 4.12 | 51.01 ± 2.21 |
| HGB | g/L | 138.51±5.51 | 131.37 ± 8.12 | 135.47 ± 5.58 |
| PLT | 10^9^/L | 480 ± 41.21 | 465 ± 36.92 | 489±32.1 |
| MCV | fL | 48.85 ± 2.1 | 50.1 ± 0.78 | 47.74 ± 1.65 |
| MCHC | g/L | 261.22 ± 8.8 | 253 ± 4.67 | 271.64 ± 4.22 |
| MCH | pg | 13.15 ± 0.32 | 13.75 ± 0.4 | 12.25 ± 0.33 |

Nine Balb/c mice were divided into three groups randomly, and received different treatments with the tail vein injections of nothing (control), PBS (200 μL) and NMOF-545@Pt (200 μL, 10 mg/kg), separately. All mice were sacrificed after two weeks of injection, and the blood was collected for biochemistry tests. White blood cell (WBC), red blood cell (RBC), hematocrit (HCT), hemoglobin (HGB), platelets (PLT), mean corpuscular volume (MCV), mean corpuscular hemoglobin concentration (MCHC), mean corpuscular hemoglobin (MCH).
